# Effect of Dietary Tyrosine on Behavior and Ruminal Meta-Taxonomic Profile of Altay Sheep with Different Temperaments

**DOI:** 10.3390/vetsci12080684

**Published:** 2025-07-22

**Authors:** Asmita Thapa, Weidong Huang, Shahab Ur Rehman, Muhammad Hammad Zafar, Jinying Zhang, Luoyang Ding, Mengzhi Wang

**Affiliations:** 1 Laboratory of Metabolic Manipulation of Herbivores Animal Nutrition, College of Animal Science and Technology, Yangzhou University, Yangzhou 225009, China; 2 School of Agriculture and Environment (M087), University of Western Australia Institute of Agriculture, The University of Western Australia, Crawley, WA 6009, Australia

**Keywords:** stress, tyrosine, rumen microbiota, animal behavior, Altay sheep

## Abstract

The objective of this study was to evaluate the effect of tyrosine on behavior and ruminal meta-taxonomic profile of Altay sheep. Twelve rams with the lowest behavioral responses to humans and twelve rams with the highest behavioral responses were sorted into calm and nervous groups, respectively. Following the 2 × 2 factorial design, the rams from each group were equally assigned two treatments of a basal diet and a diet with an extra 4 g of tyrosine for 30 d. The temperament trait, growth performance, rumen fermentation parameters, ruminal epithelium antioxidant capacity, and rumen microbial composition were measured at the end of the feeding experiment. The results showed that tyrosine treatment led to a decreased number of crosses in the arena test in nervous sheep (*p* < 0.05). The tyrosine treatment led to increased antioxidant markers in the rumen epithelium, such as catalase, total antioxidant capacity, and GSH content in rumen epithelial tissues (*p* < 0.05) in calm and nervous sheep. The total volatile fatty acids and propionic acid content in the rumen were increased by tyrosine treatment in nervous and calm sheep (*p* < 0.05). The rumen microbial study revealed that the dominant microbial genera were *Cryptobacteroides*, *Prevotella*, *Limivicinus*, *Quinella*, *UBA1711*, *RUG740*, *Sachharofermentans*, *Limomorpha*, *Soladiphilus*, *Flexinia*, and others in both the tyrosine treatment and temperament groups. A combined effect of treatment and temperament was seen on the abundance of two microbial genera, *UBA1711* and *RUG740* (*p* < 0.05). These findings suggest that tyrosine-treated Altay sheep would experience less stress during production, resulting in reduced oxidative stress in the rumen epithelium and improved propionic acid production in the rumen compared with that of basal-diet-treated Altay sheep. Meanwhile, the effect of the dietary tyrosine treatment on ruminal microbial diversity was lower, suggesting lesser degradation of tyrosine by ruminal microbes in Altay sheep.

## Simple Summary

This study investigated the effects of tyrosine supplementation on behavior and ruminal microbiota in Altay sheep, categorized by temperament (calm vs. nervous). Sheep were fed either a basic diet or a tyrosine-supplemented diet for 30 days. The results showed that tyrosine reduced anxiety-like behaviors in nervous sheep and altered rumen fermentation, notably increasing propionic acid levels. Although temperament did not significantly impact microbial diversity, tyrosine supplementation influenced the abundance of certain microbial genera. Additionally, tyrosine improved the antioxidant capacity of the rumen epithelium, particularly in nervous sheep. These findings suggest that tyrosine could be a beneficial feed additive to reduce stress and enhance ruminal health in sheep, with potential applications in intensive farming systems.

## 1. Introduction

During the last few decades, new intensive production systems have been practiced in livestock production. Intensive farming focuses on maximizing animal production with limited space and resources, often involving high stocking densities and regulated environments to expedite growth and yield. Because of this, physiological and psychological stresses on animals have increased [1,2]. These stressors directly or indirectly affect the health and productivity of animals [3]. Animal temperament is defined by the behavioral responses of animals towards stress. It can be evaluated by assessing behavioral and personal traits [4,5]. Less reactive animals (i.e., calm animals) show higher productivity than more reactive animals (i.e., nervous animals). Nervous cattle have more bruises on their carcasses than calm cattle [6,7]. Calm dairy cows are easier to handle during milking, reducing milking time and increasing milk yield [8,9,10]. Nervous pigs show more aggressive behavior and more bruises on their carcasses [11,12]. Docile ewes show better maternal behavior and increased lamb survival rates [13,14]. Calm animals have better meat quality and productivity [15,16,17,18,19]. Calm animals have a better antioxidant capacity than nervous ones [20]. This suggests that calm animals might be more beneficial for intensive farming systems than nervous ones.

Tyrosine is a biochemical precursor of the catecholamines dopamine and norepinephrine [21]. The enzyme tyrosine hydroxylase in the body transforms dietary L-tyrosine into L-DOPA, the direct precursor of dopamine, which is then transformed to norepinephrine [22]. Tyrosine supplementation has been suggested as a possible remedy for performance issues brought by stress [23]. Tyrosine can enhance DA (dopamine) and NE (norepinephrine) levels in the brain, and this possibility has led numerous studies to investigate whether tyrosine can positively influence cognitive or behavioral performances that rely on catecholamine function [23]. Tyrosine has been used in the treatment of different human diseases such as schizophrenia [24], attention-deficit hyperactivity disorder (ADHD) [25], Parkinson’s disease [26,27], and phenylketonuria [28,29]. But its effect is not fully understood. Some results show its effectiveness in improving the symptoms of diseases, but other results show no effect. In healthy humans, tyrosine supplementation has been found to improve working memory when there is not much stress [30]. Furthermore, tyrosine supplementation has improved performance in DA-driven cognitive tests [31], improved the exposure towards cold stimuli [32], and reduced environmental stress [33]. Tyrosine’s effect on animals is less studied. In pigs, the addition of tyrosine was beneficial for improving meat quality and reducing nitrogen emissions [34]. Some research suggests that tyrosine might be a good feed additive for broilers [35,36]. Furthermore, tyrosine has improved the stress response in different fish species [37,38]. Overall, these findings suggest that tyrosine holds promise as a feed additive for reducing stress.

The rumen is an anaerobic chamber of ruminants and consists of various microbes. Ruminal microbes play a crucial role in converting plant components of the diet into energy into volatile fatty acids [39,40]. Ruminant microbes are affected by various factors such as the diet [41,42], age [43], weaning [44,45], disease status [46,47], stress [48,49], and temperament [19] of animals. Microbes can produce different metabolites, and such metabolites can alter the metabolic functions of ruminants. More than 70% of VFAs are absorbed through the rumen epithelial wall and utilized by the animal [50]; therefore, ruminal epithelial health is important for animal health and production [51]. When the ruminal environment changes, ruminal oxidative stress may be triggered, which eventually triggers ruminal epithelial wall inflammation and damage [52]. This might include changes in nutrient contents, an increase in the number of pathogens, the invasion of pathogenic bacilli, mycotoxin contamination, and heavy metal contamination. These factors upset the balance of gut microbes, which leads to an increase in harmful microflora in the rumen. This can trigger ruminal epithelium wall inflammation and damage [53]. Overall, ruminal microbial diversity and ruminal epithelial health are important for ruminants.

Interest in interactions among the gut microbiome, behavior, and the brain has increased. The role of gut microbiome in behavioral responses is associated with pain, emotion, social interactions, and feed intake in humans. The gut–brain axis is bidirectional communication [54]. Alterations of microbiota have been suggested in the pathophysiology of various brain disorders, including disorders of mood and affect [55], autism spectrum disorders [55], Parkinson’s disease [56], schizophrenia [57], and chronic pain [58] in humans. In farm animals, the microbial components of the gut–brain axis also influence the behavior (feeding, anxiety-like, social) and memory [59] of animals, though studies on this subject are smaller in number. Therefore, the aim of our study was to observe the tyrosine’s effects on stress-related behavior in Altay sheep and its possible effects on rumen fermentation parameters and rumen microbial structures, along with the antioxidant parameters of the rumen epithelium.

## 2. Materials and Methods

### 2.1. Selection for Temperament Using Arena Test of Behavior

A total of 200 six-month-old Altay rams underwent behavioral testing via the arena test. Based on the frequency of crosses, 12 calm and 12 nervous individuals were selected.

Arena test of behavior:

It has been demonstrated that an arena test intended to induce approach/avoidance motivational conflict in sheep can identify variations in stimulus aversiveness. Presenting a stimulus to the individual test sheep and a group of companions causes approach/avoidance conflict [60]. A 7 × 3 m closed arena was divided into 4 equal parts. The test sheep entered the first part of the arena through a gate. At the end of the fourth part of the arena, there was a pen containing a group of sheep. But a static human was standing in front of that pen. While sheep exhibit a natural preference for rejoining their flock, the presence of a human obstructing access elicits variable behavioral responses among individuals. The number of crosses the test sheep made in each part of the arena within 3 min was measured. Sheep with more crosses were considered nervous, while those with fewer crosses were considered calm. The behavioral test was conducted twice: first, before the start of the animal trial, and second, after the completion of the feeding period, i.e., following tyrosine supplementation in the experimental group.

### 2.2. Animal Feeding and Management

Each sheep was housed in an individual pen (1.25 m × 1 m) for the trial. After an adjustment period of 7 days, animals were housed for 30 days. On the basis of a 2 × 2 factorial method design, calm sheep were randomly divided into two groups: 6 calm sheep were fed a basic diet, and the remaining 6 sheep were supplemented with 4 g of tyrosine per day with a basic diet. Similarly, 6 nervous sheep were fed a basic diet, and 6 nervous sheep were supplemented with 4 g of tyrosine per day along with a basic diet. Tyrosine was purchased from Shanghai Yuanye Biological Co., Ltd. (Shanghai, China) and had a purity of 99%. Animals were fed twice a day at 10:00 and 18:00, while water was available ad libitum. Details of the basal diet composition and nutrient content are provided in Table 1. 

Each kilogram of the premix contained the following: Cu: 25 mg/kg, Fe: 75 mg/kg, Zn: 105 mg/kg, Cocl2: 0.0024 mg/kg, Na: 0.016 mg/kg, VA: 12,000 IU/kg, VD: 310,000 IU/kg, VE: 25 mg/kg, nicotinic acid: 36 mg/kg, choline: 1000 mg/kg.

After 30 days, the behavior of the rams was assessed using the arena test, during which the number of crosses was recorded. Following the second behavioral test, the sheep were transported to the slaughterhouse for slaughter. In accordance with standard experimental protocols, the animals were fasted for 24 h prior to slaughter.

### 2.3. Rumen Sample Collection and Processing

In slaughter sampling, after the removal of internal organs and the intestine, fresh rumen samples were collected within 10 min of slaughtering. Rumen fluid contents were filtered through four layers of sterilized cotton gauze. Filtered rumen fluid was collected in a 10 mL tube and quickly stored at −20 °C. Then, 50 mL of unfiltered rumen contents was stored in liquid nitrogen for further analysis [61,62]. The ventral part of the rumen epithelium was also collected and stored at −80 °C for further analysis.

### 2.4. Measurement Indices and Methods

#### 2.4.1. Determination of Growth Performance Indicators

The sheep were measured at the start of the experiment, day 1 (initial weight), and end of the experiment, day 30 (final weight). For consistency, sheep were weighed at the same time of day (before feeding). The average daily gain (ADG) was measured using the formula

(1) $$\mathrm{ADG}=\frac{\mathrm{Final}\;\mathrm{weight}\;-\;\mathrm{Initial}\;\mathrm{weight}}{\mathrm{number}\;\mathrm{of}\;\mathrm{feeding}\;\mathrm{days}\;(30\;d)}$$

The total amount of feed (kg) given to sheep over the trial period was recorded. Every morning, before the sheep were fed, the leftover sheep feed was measured. The actual consumption of feed was calculated by subtracting the leftover feed from the total feed given. The total feed conversion ratio (FCR) was calculated by using the formula

(2) $$\mathrm{FCR}=\frac{\mathrm{Feed}\;\mathrm{consumed}\;(\mathrm{kg})}{\mathrm{weight}\;\mathrm{gain}\;(\mathrm{kg})}$$

#### 2.4.2. Determination of Rumen Fermentation Parameters

Volatile fatty acid concentration was measured in a metabolic manipulation laboratory using the gas chromatography method. After the rumen contents were thawed at 4 °C, centrifugation was performed (12,000× *g* for 5 min). Then, 0.2 mL of 20% metaphosphoric acid containing 60 mM crotonic acid was added to 1 mL of the supernatant obtained from centrifugation. The obtained mixture was filtered through a 0.22 m needle filter. After that, a 1 microliter sample was taken and inserted into a spectrometer using a syringe. Then, the concentration of volatile fatty acids was determined using a regression equation with a standard curve [63].

#### 2.4.3. Determination of Rumen Epithelium Antioxidant Properties

Antioxidant indices (total antioxidants (T-AOC), catalase (CAT), glutathione (GSH), glutathione peroxidase (GSH-Px), superoxide excretion enzyme (SOD), and malondialdehyde (MDA) were determined. SOD activity was determined by the xanthine oxidase method, MDA content was determined by the thiobarbituric acid method, T-AOC was determined by the total antioxidant capacity detection kit (ABTS method), and GSH-Px activity and CAT activity were determined by the colorimetric method. All the kits used for index determination were purchased from Beijing Solarbio Science and Technology Co., Ltd. (Beijing, China) and tested according to the kit instructions.

#### 2.4.4. Determination of Tyrosine Content in Rumen

The extraction and determination of tyrosine in rumen fluid were conducted by an amino acid analyzer (LA8080, Hitachi, Tokyo, Japan) according to “Determination of Amino Acids in Food” [64]. This was carried out by Shanghai Ling En Biotech Co., Ltd. (Shanghai, China). First, 1 mL of a sample was taken into a hydrolysis tube, and the volume was adjusted to 10 mL with 6 mol/L hydrochloric acid solution. Next, four drops of phenol were added to the hydrolysis tube. The tube was then placed in a refrigerant and frozen for 3 min before being connected to a vacuum pump. A vacuum was applied (close to 0 Pa), and the tube was subsequently filled with nitrogen. This vacuum and nitrogen-filling process was repeated three times. The sealed hydrolysis tube was placed in an electro-thermal constant temperature blast chamber, hydrolyzed for 22 h, removed, and cooled to room temperature. The hydrolysis tube was then opened, and the hydrolysate was filtered using a 0.45 μm aqueous membrane. An accurate volume of 1.0 mL of the filtrate was taken, and a parallel evaporator was used under reduced pressure at a temperature of 40–50 °C. Lastly, the sample was redissolved with 1 mL amino acid dilution solution, passed through a 0.22 μm filter membrane, and transferred to the instrument injection bottle for sample determination [64]. 

#### 2.4.5. DNA Extraction and Metagenomics

DNA extraction and metagenomics sequencing were performed by Linen Biotech (Shanghai, China). Total DNA was extracted from ruminal fluid samples using the E.Z.N.A.^®^ Viral DNA Kit (Omega Bio-tek, Norcross, GA, USA) according to the manufacturer’s protocols. A high-quality DNA sample (OD260/280 = 1.8~2.2, OD260/230 ≥ 2.0) was used to construct a sequencing library. Metagenomic shotgun sequencing libraries were constructed and sequenced at Shanghai Biozeron Biological Technology Co., Ltd. (Shanghai, China). In brief, for each sample, a TruSeq DNA Library Preparation kit (catalog No.: FC-121-2001, Illumina, San Diego, CA, USA) was used to construct sequencing libraries, and the concentrations of all libraries were measured by a High Sensitivity Double Stranded DNA kit on a Qubit Flurometer (Thermo Fisher Scientific, Waltham, MA, USA). All samples were sequenced in the NGS instrument with the paired-end 150 bp (PE150) mode.

### 2.5. Statistical and Abundance Analysis

Behavioral data were sorted out by Excel2016. After that, IBM SPSS Statistics 26.0.0.3 software was used to analyze data, using the Univariate program and the General Linear Model module. Multiple comparisons between groups were performed using the Bonferroni method. All the graphs were created using GraphPadPrism 9.0 and Origin 2022 software. The results are presented as the mean and SEM. Significance was considered when *p* ≤ 0.05.

## 3. Results

### 3.1. Effect of Tyrosine on Growth and Feed Utilization of Altay Sheep with Different Temperaments

**Figure 1:**
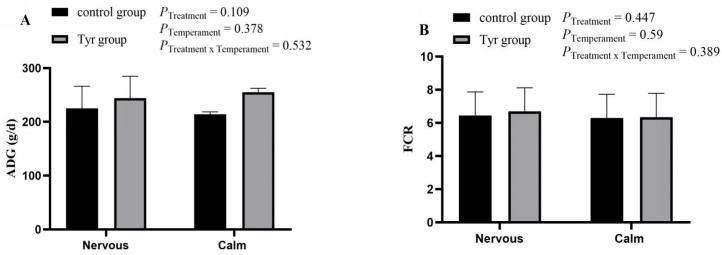
Effect of tyrosine on feeding behavior in calm and nervous sheep. (**A**) ADG; (**B**) FCR. *n* = 6, black bar represents control group, brown bar represents tyrosine group. Data are expressed as mean ± SEM.

There was no significant difference in the ADG of the calm and nervous groups as well as the tyrosine-supplemented and normal diet groups (*p* > 0.05, Figure 1A). The ADG of calm sheep with a basic diet was 214.11 g/d, and that of tyrosine-supplemented calm sheep was 255.23 g/d. Similarly, nervous sheep with a basic diet had an ADG of 224.95 g/d, while tyrosine-treated nervous sheep had 244.195 g/d. There was no significant difference seen in the FCR between both the temperament and treatment groups (*p* > 0.05, Figure 1B). The FCR of calm sheep with a basic diet was 6.3, and that of tyrosine-treated calm sheep was 6.35. Similarly, the FCR of nervous sheep with a basic diet was 6.45, and that of tyrosine-treated nervous sheep was 6.7 (Figure 1B; Supplementary Table S1).

### 3.2. Effect of Tyrosine on Behavior of Altay Sheep with Different Temperaments

**Figure 2:**
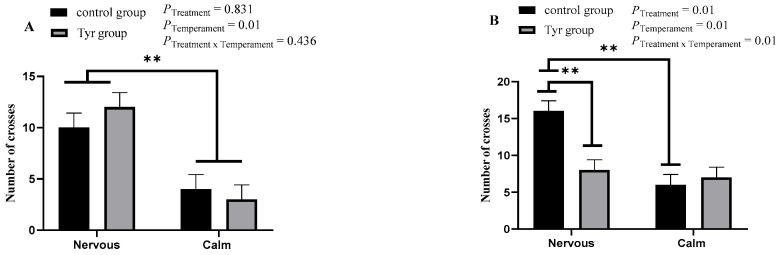
The effect of tyrosine on the number of crosses shown by calm and nervous sheep in the arena test of behavior. *n* = 6, black bar represents control group, brown bar represents tyrosine group. (**A**) Test 1, on day 0; (**B**) test 2, on day 30. Data are expressed as mean ± SEM. **: *p* < 0.01.

Figure 2 represents the effect of tyrosine on the number of crosses in the arena test of behavior. Sheep from the calm group had a significantly lower number of crosses than the nervous group (*p* < 0.01) at the first arena test (Figure 2A). Similarly, in the second arena test, calm sheep exhibited a significantly lower number of crosses compared to nervous sheep (*p* < 0.01; Figure 2B). A significant reduction in the number of crosses was also observed in nervous sheep that received tyrosine supplementation. The number of crosses shown by tyrosine-treated nervous sheep was 8, while the number of crosses shown by basic-diet-fed nervous sheep was 16 (Figure 2B; Supplementary Table S2). On the other hand, tyrosine supplementation increased the number of crosses shown by calm sheep. Overall, a combined interaction of temperament and tyrosine supplementation was seen in the second arena test (*p* < 0.05, Figure 2B).

### 3.3. Rumen Fermentation Parameters

**Figure 3:**
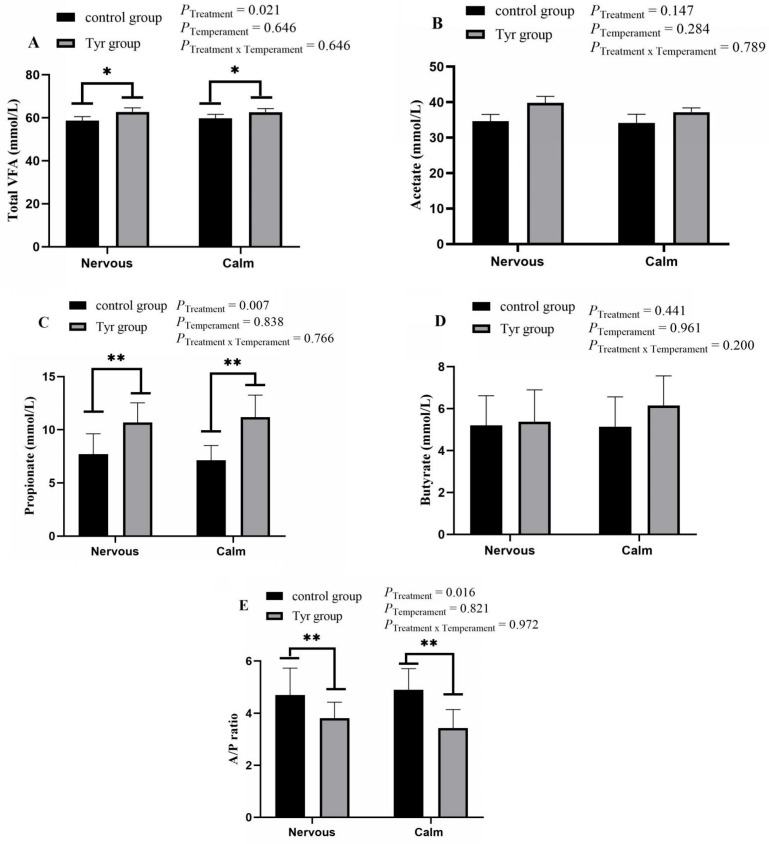
Effect of tyrosine on rumen fermentation parameters of Altay sheep with different temperaments. *n* = 6, black bar represents control group, brown bar represents tyrosine group. (**A**) Total volatile fatty acids (TVFAs), (**B**) acetate, (**C**) propionate, (**D**) butyrate, (**E**) A/P ratio. Data are expressed as mean ± SEM. *: *p* < 0.05, **: *p* < 0.01.

The total volatile fatty acids and major volatile fatty acids present in rumen fluid are presented in Figure 3. No interaction effect between temperament and tyrosine supplementation was seen on the rumen fermentation parameters in this study (*p* > 0.05). There was a significant difference seen in total volatile fatty acids (TVFAs) in the tyrosine-treated calm and nervous groups (Figure 3A; Supplementary Table S3). Tyrosine supplementation significantly increased the total volatile fatty acid concentration in calm and nervous sheep. The TVFA concentration of nervous sheep with a basic diet was 58.73 mmol/L, and that of tyrosine-treated nervous sheep was 62.74 mmol/L. The TVFA concentration was 59.85 mmol/L in basic-diet-fed calm sheep and 62.62 mmol/L in tyrosine-supplemented calm sheep. There was no significant difference seen in the amount of acetate in the temperament and tyrosine treatment groups (Figure 3B). The concentration of propionic acid was significantly increased in the tyrosine supplement groups (Figure 3C). No significant differences in rumen butyrate concentrations were observed between temperament groups or tyrosine treatment groups (Figure 3D). Tyrosine treatment significantly decreased the A/P (acetate/propionate) ratio in both nervous and calm sheep (Figure 3E).

### 3.4. Tyrosine Content in Rumen Fluid

**Figure 4:**
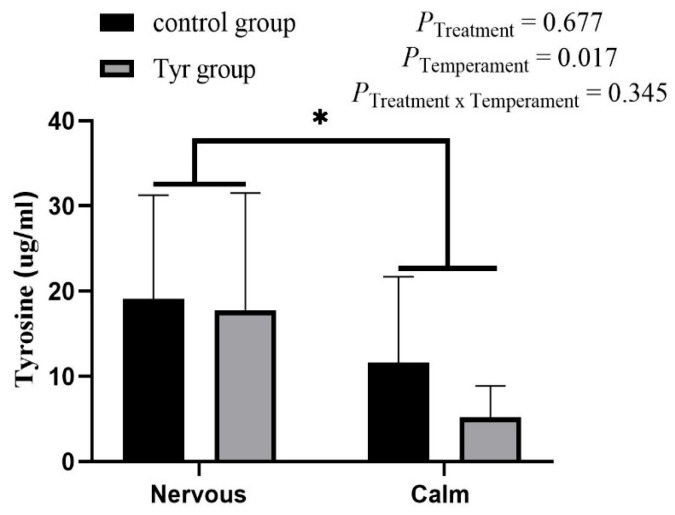
Tyrosine content in rumen fluid of Altay sheep. *n* = 6, black bar represents control group, brown bar represents tyrosine group. Data are expressed as mean ± SEM. *: *p* < 0.05.

Figure 4 represents the tyrosine content in rumen fluid. The results show that there was no significant difference seen in tyrosine amount between the treatment groups. There was a significant difference between the temperament groups. The nervous groups had a significantly higher amount of tyrosine than the calm groups. No interaction effect between temperament and tyrosine was seen on the amount of tyrosine in rumen fluid (Figure 4; Supplementary Table S4).

### 3.5. Antioxidant Properties of Rumen Epithelium Tissues

**Figure 5:**
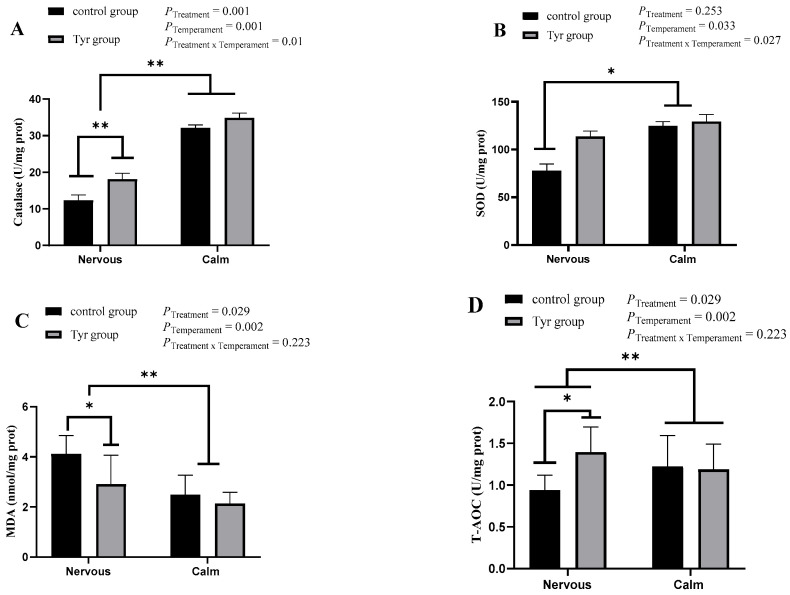

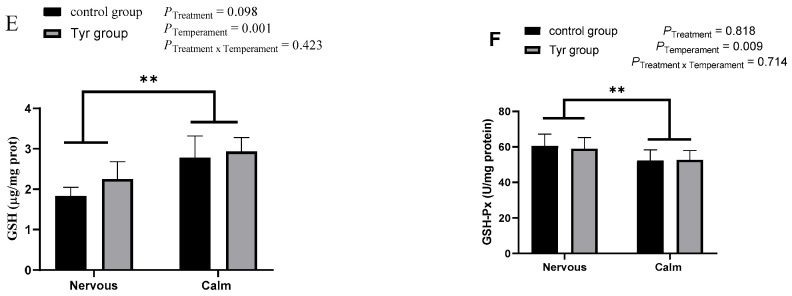
Effect of tyrosine on antioxidant properties of rumen epithelial tissues, *n* = 6, black bar represents control group, brown bar represents tyrosine group. (**A**) Catalase, (**B**) superoxide dismutase (SOD), (**C**) malondialdehyde (MDA), (**D**) total antioxidant capacity (T-AOC), (**E**) glutathione (GHS), (**F**) glutathione peroxide. Data are expressed as mean ± SEM. *: *p* < 0.05, **: *p* < 0.01.

The antioxidant properties of the rumen epithelium are presented in Figure 5. The results indicated a significant difference in catalase activity between the different temperament and treatment groups (*p* < 0.05; Figure 5A). For the SOD value, there was a significant difference between the temperament groups (*p* < 0.05, Figure 5B). A combined effect of temperament and treatment on the SOD value was also seen. The MDA value in the rumen epithelium was significantly affected by both the temperament and treatment factors (*p* < 0.05, Figure 5C). Calm sheep had a significantly lower MDA value than nervous ones (*p* < 0.01, Figure 5C). The total antioxidant capacity of rumen epithelium was affected by both temperament and treatment (*p* < 0.05, Figure 5D). There was a significant difference in the GHS value between the different temperament groups (*p* < 0.05, Figure 5E). There was a significant difference seen in the GHS-Px value between the different temperament groups (*p* < 0.05, Figure 5F; Supplementary Table S5). Nervous sheep had a significantly higher GHS-Px value than the calm ones.

### 3.6. Effect of Tyrosine on Rumen Microbial Structure of Altay Sheep with Different Temperaments

#### 3.6.1. Rumen Microbial Sequencing and Alpha Diversities

**Figure 6:**
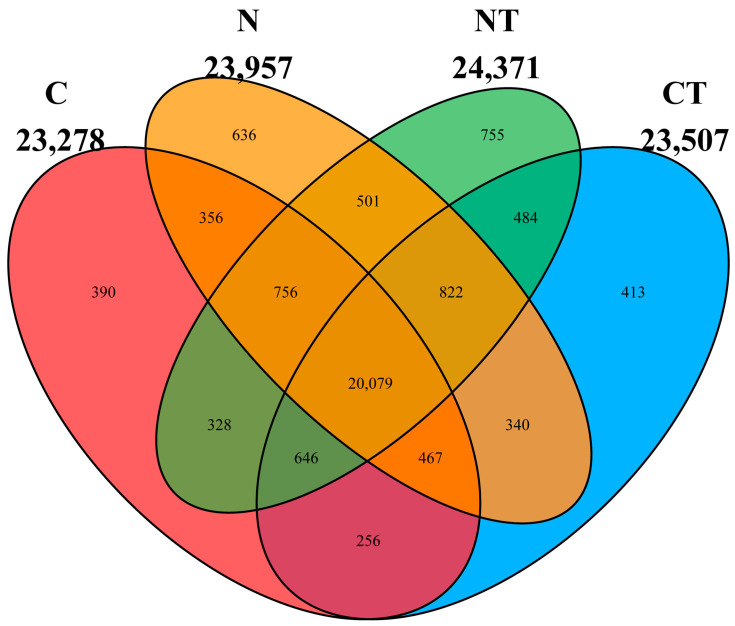
Microbial genes present in different groups. C represents calm group, CT represents calm sheep with tyrosine treatment, N represents nervous group, and NT represents nervous sheep with tyrosine treatment.

**Figure 7:**
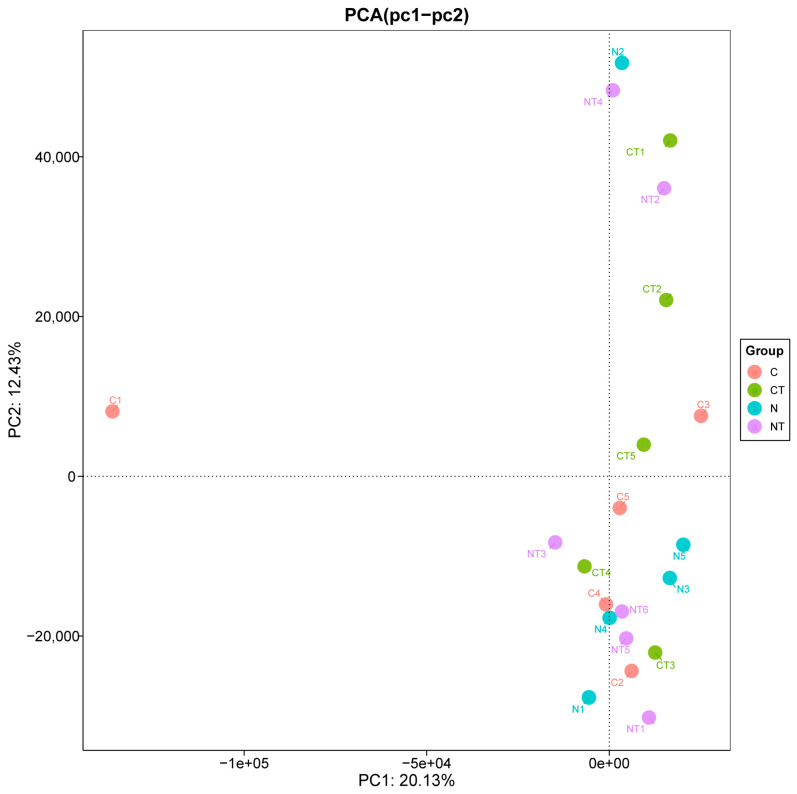
Principal component analysis of the β diversity of the microbiome in Altay sheep with different temperaments. C represents the calm group, CT represents calm sheep with tyrosine treatment, N represents the nervous group, and NT represents nervous sheep with tyrosine treatment.

Figure 6 represents the number of rumen microbial genes present in different groups. Calm and nervous sheep fed with a basic diet had 23,278 and 23,957 microbial genes, respectively. In contrast, the tyrosine-supplemented calm and nervous groups had 23,507 and 24,371 microbial genes, respectively. In total, 20,079 genes were seen to be common in all the groups. Out of 23,278 genomes present in the calm group, only 390 genomes were unique. Out of 23,957 genomes present in the nervous group, 636 genomes were unique. There were 755 genomes unique to the nervous tyrosine group and 413 genomes unique to the calm tyrosine group. There was a significant difference in richness at the genus level in the temperament group (*p* = 0.046). There was no significant difference in Shannon’s and Simpson’s indices between the temperament and treatment groups at the genus level (Table 2). A principal component analysis of β diversity showed that there were no significant differences in bacterial composition between the groups (Figure 7).

#### 3.6.2. Effect of Tyrosine on Relative Abundance of Rumen Bacterial Community at Phylum and Genus Levels

**Figure 8:**
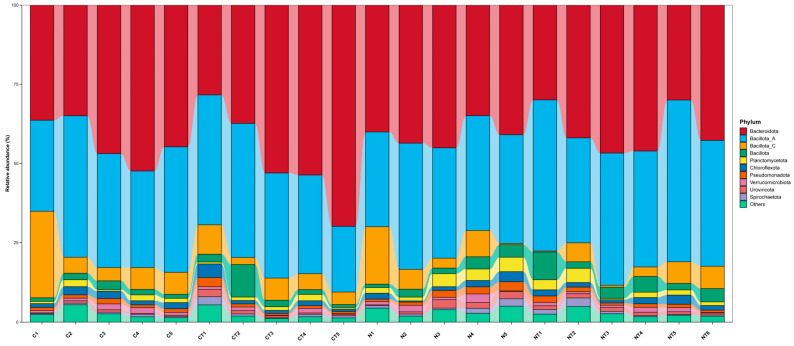
The effect of tyrosine on the composition of rumen bacteria at the phylum level in Altay sheep with different temperaments. C represents the calm group, CT represents calm sheep with tyrosine treatment, N represents the nervous group, and NT represents nervous sheep with tyrosine treatment.

**Figure 9:**
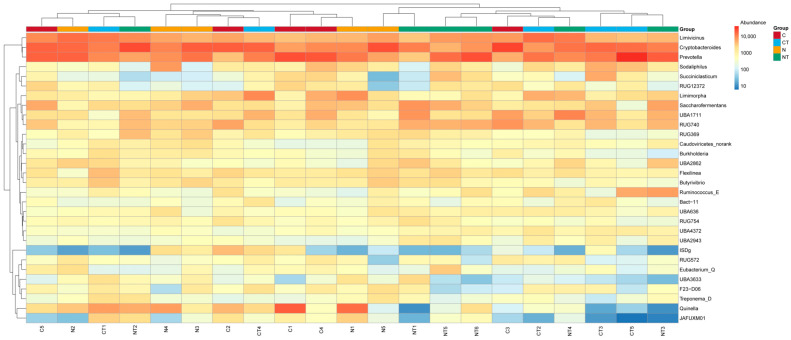
The effect of tyrosine on the composition of rumen bacteria at the genus level in Altay sheep with different temperaments. C represents the calm group, CT represents calm sheep with tyrosine treatment, N represents the nervous group, and NT represents nervous sheep with tyrosine treatment.

Figure 8 represents the dominant microbial phyla present in the rumen fluid of the Altay sheep. On the basis of taxonomic analysis, the dominant members of the bacterial community at the phylum level were *Bacteriodota*, *Bacillota A*, *Bacillota C*, *Bacillota*, *Planctomycetota*, *Chloroflexota*, *Pseudomonadota*, and *Verrucomicorbiota*. At the genus level, the dominant groups were *Crptobacteroides*, *Prevotella*, *Limivucinus*, *Quinella*, *UBA1711*, *RUG740*, *Sachharofermentans*, *Limomoroha*, *Soladiphilus*, *flexinia*, and others (Figure 9; Supplementary Table S6).

At the phylum level, the relative abundance of *Planctomycetota* was significantly higher in the nervous group (*p* < 0.05; Table 3). There was no significant difference in the relative abundance of *Planctomycetota* between the tyrosine treatment groups. There were no significant differences seen in the relative abundance of *Bacteroidota*, *Bacillota A*, *Bacillota C*, *Chloroflexota*, *Pseudomonadota*, and *Verrucomicrobiota* between both the temperament and treatment groups (*p* > 0.05).

At the genus level, there were no significant differences in the relative abundance of *Cryptobacteroides*, *Prevotella*, *Limivicinus*, *Quinella*, *Schharofermentans*, *Limimorpha*, *Sodaliphilus*, *Flexinia*, and others between both the temperament and treatment groups (*p* >0.05; Table 4). But the abundance of two genera, *UBA1711* and *RUG740*, was affected by both factors, temperament and treatment (*p* < 0.05; Table 4).

### 3.7. Prediction of Gene Functions Through KEGG

**Figure 10:**
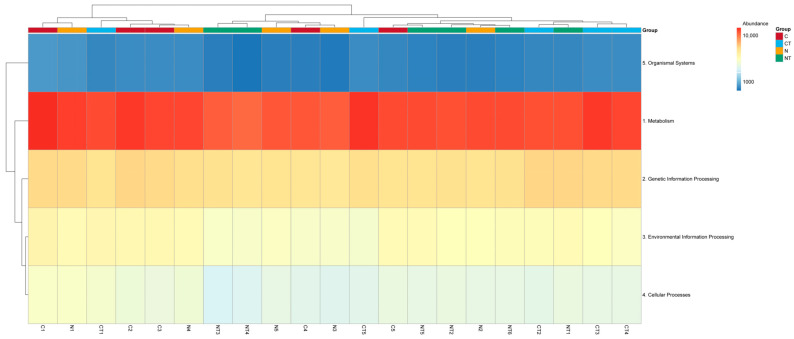
Clustered heat map showing microbial function prediction through KEGG pathway level 1. C represents calm group, CT represents calm sheep with tyrosine treatment, N represents nervous group, and NT represents nervous sheep with tyrosine treatment.

**Figure 11:**
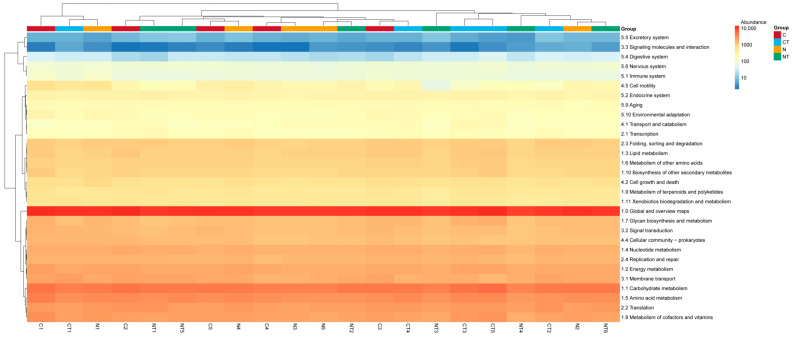
Clustered heat map showing microbial function through KEGG pathway level 2. C represents calm group, CT represents calm sheep with tyrosine treatment, N represents nervous group, and NT represents nervous sheep with tyrosine treatment.

**Figure 12:**
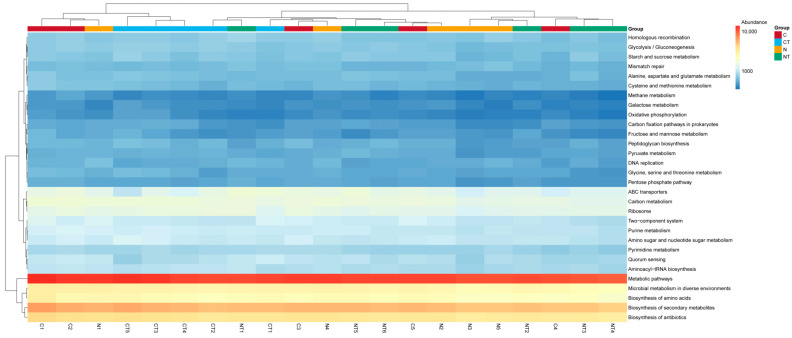
Clustered heat map showing microbial function prediction through KEGG level 3. C represents calm group, CT represents calm sheep with tyrosine treatment, N represents nervous group, and NT represents nervous sheep with tyrosine treatment.

The predictions of bacterial gene functions from KEGG pathway levels 1, 2, and 3 are shown in Figure 10, Figure 11 and Figure 12, respectively. On level 1, the abundant genes identified were related to metabolism and genetic information processing. On KEGG pathway level 2, genes related to global and overview maps, carbohydrate metabolism, amino acid metabolism, signal transduction, and energy metabolism were abundant (Figure 11; Supplementary Table S7). On KEGG pathway level 3, genes related to metabolic pathways, the biosynthesis of secondary metabolites, and the biosynthesis of antibiotics were predicted to be in greater abundance in all samples (Figure 12).

On KEGG pathway level 1, there was a significant difference in pathways related to metabolism between the temperament groups (*p* < 0.05; Table 5). The calm sheep had a higher number of genes related to metabolism than the nervous ones. There was no significant difference seen in gene function prediction in other pathways between both the temperament and tyrosine groups (*p* > 0.05; Table 5).

On KEGG pathway level 2, there was a significant difference seen between the temperament groups for different pathways. Pathways related to global and overview maps; carbohydrate metabolism; energy metabolism; lipid metabolism; amino acid metabolism; metabolism of other amino acids; glycan biosynthesis and metabolism; metabolism of terpenoids and polyketides; and folding, sorting, and degradation had significance differences between the calm and nervous groups (*p* < 0.05; Table 6). The calm group had a significantly higher number of genes than the nervous one. Gene function related to cellular community (eukaryotes) had a significant difference between the tyrosine treatment groups (*p* < 0.05; Table 6). Tyrosine treatment had reduced the number of genes in both the nervous and calm groups.

On the basis of KEGG pathway level 3, there was a significant difference in gene function related to metabolic pathways, the biosynthesis of secondary metabolites, the biosynthesis of carbon metabolism, amino sugar and nucleotide sugar metabolism, thyroid hormone synthesis, and oxidative phosphorylation between the different temperament groups (*p* < 0.05; Table 7). There was no significant difference in the above pathways between the treatment groups. For gene function related to the two-component system, there was a significant difference between the tyrosine treatment groups (*p* = 0.05; Table 7). There was no significant difference in tyrosine metabolism and phenylalanine, tyrosine, and tryptophan biosynthesis function between both the temperament and treatment groups (*p* > 0.05; Table 7).

### 3.8. Correlation Analysis Between Abundant Microbiota, Rumen Epithelium Antioxidant Properties, and Abundant Functional Pathways

**Figure 13:**
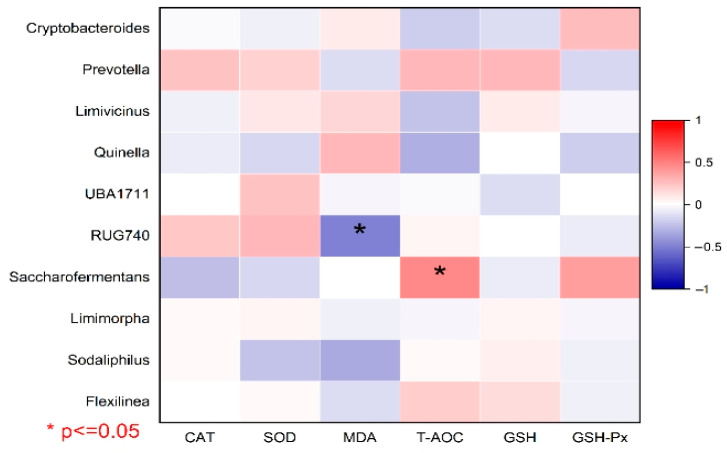
Correlation analysis between abundant microbial genera and rumen epithelium antioxidant properties. A (+1) value represents a positive correlation, and a (−1) value represents a negative correlation. Correlation was considered significant when *p* ≤ 0.05.

Figure 13 shows a correlation analysis between the abundant microbial genera and rumen epithelium antioxidant properties. According to the results, the total antioxidant capacity of the rumen was positively correlated with *Saccharofermentans* (*p* < 0.05, Figure 13). On the other hand, the MDA concentration was negatively correlated with the *RUG740* bacterial genus.

**Figure 14:**
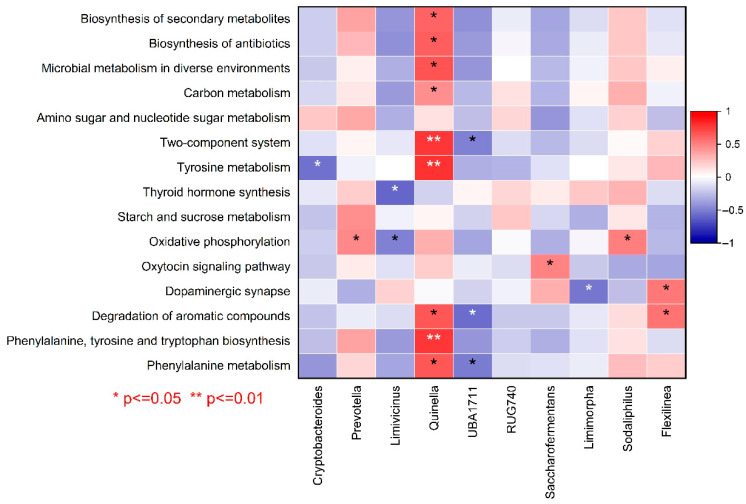
Correlation analysis between abundant microbial genera and metabolic pathways. A (+1) value represents a positive correlation, and a (−1) value represents a negative correlation. Correlation was considered significant when *p* ≤ 0.05. *: *p* ≤ 0.05, **: *p* ≤ 0.01.

Figure 14 shows the correlation analysis between abundant microbial genera and metabolic pathways on the basis of KEGG functional analysis. The results show that *Quinella* was positively correlated with phenylalanine, tyrosine, and tryptophan biosynthesis (*p* < 0.05, Figure 14). It was also found to be positively correlated with tyrosine metabolism and the two-component system. The bacterial genus *UBA1711* was negatively correlated with phenylalanine metabolism, the degradation of organic compounds, and the two-component system. Furthermore, the abundance of *Cryptobacteroides* was negatively correlated with tyrosine metabolism.

## 4. Discussion

The aim of this study was to analyze the effect of tyrosine on the behavior and rumen microbial structure of Altay sheep assigned by temperament. Our results suggest that there is a strong interaction between tyrosine and the behavior of Altay sheep. But the effect of tyrosine on rumen microbial structure and function was found to be weaker. A decrease in the number of crosses in the second behavior test of nervous sheep was observed in our study. In addition, the addition of tyrosine increased the propionic acid concentration in the rumen. Tyrosine addition reduced the rumen epithelium antioxidant properties of sheep.

The feeding behavior of sheep was unchanged in calm and nervous sheep as well as tyrosine-treated sheep. Though tyrosine treatment increased the ADG and FCR to some extent in both the calm and nervous groups, this increase was not at a significant level. This might be due to the short feeding period of one month. The addition of tyrosine appeared to alter stress-related behavior in sheep. The number of crosses in nervous sheep significantly decreased after tyrosine supplementation. This might be because tyrosine might have helped in the production of dopamine in the brain, and dopamine helped the sheep to cope with the stress response in the second behavior test [65]. Tyrosine counteracts stress-induced cognitive deficits and reduces environmental stress in humans, which aligns with the findings in our study regarding tyrosine’s role in reducing stress-related behaviors [23,33]. Similarly, tyrosine supplementation decreased stress during the transportation of desert sheep and stress-related parameters in fish [37,38,66], which also correlates with our findings.

The rumen function of sheep was different in the treatment groups, as the addition of tyrosine increased the propionic acid concentration in both nervous and calm sheep. But the acetic acid concentrations were similar. Therefore, there was a greater ratio of acetic to propionic acid. The change in ratio of acetic to propionic acid is related to microbial structures in the rumen [67]. It indicates that there was a change in the rumen fermentation mode towards propionic acid fermentation in both tyrosine-treated groups. More rumen microbial genes were found in the tyrosine treatment groups, which correlated with different rumen fermentation parameters in the tyrosine treatment groups.

Tyrosine is degraded by rumen bacteria and forms microbial proteins like other amino acids [68]. In our study, nervous sheep had a higher amount of tyrosine in rumen fluid than calm ones. This shows that tyrosine is less degraded in the rumen of nervous sheep than in that of calm ones. Dietary tyrosine is first degraded in the rumen; the rest reaches the small intestine for absorption [69]. Therefore, in our study, tyrosine might have moved to the small intestine and been absorbed there so that it became a substrate for dopamine production in the brain [70].

The catalase content of the rumen epithelium of sheep was affected by both temperament and treatment. Both the calm and the nervous groups showed higher catalase levels after tyrosine therapy. Similarly, providing tyrosine to anxious sheep raised their SOD concentration. Tyrosine addition decreased the MDA content of nervous sheep. Generally, stressed animals have higher MDA contents [20]. The total antioxidant capacity of nervous sheep was increased due to tyrosine addition. But there was no effect of tyrosine addition on the GSH and GSH-Px values of the rumen epithelium. Tyrosine was found to improve oxidative stress [71,72]. Overall, these findings imply that tyrosine supplementation enhanced the rumen epithelium’s antioxidant capacity and the general health of sheep.

The bacterial populations were similar between the two temperament groups and the two treatment groups. The similarity observed in both temperament groups might suggest that there is no effect of temperament on the rumen microorganisms of Altay sheep, which contradicts a finding for Hu sheep [19]. This might be due to the species of sheep being different, since microbial structures can differ in different sheep breeds [73]. Furthermore, it also suggests that tyrosine might not have an effect on rumen microorganisms. The bacterial populations were similar in diversity (Shannon index) and richness (Simpson index) in both the temperament and treatment groups.

Tyrosine in the rumen is degraded by ruminal microorganisms. The tyrosine degradation ability of rumen bacteria is about 1.5 times higher than that of rumen protozoa [74]. The similarity in the relative abundance levels of the most dominant phyla, *Bacteroides*, *Bacillota A*, *Bacillota C*, and *Chloroflexota*, suggests that temperament and tyrosine might not have a direct link with the abundance of the microbiota in Altay sheep. Similarly, the most dominant genera, *Cryptobacteroides*, *Prevotella*, *limivicinus*, *Quinella*, *UBA1711*, *RUG740*, and *Saccharofermentans*, were not significantly different in abundance in both the temperament and treatment groups. But a combined effect of temperament and tyrosine on the abundance of *UBA1711* and *RUG740* was seen. The bacterial genus *UBA1711* falls under the phylum *Bacteroidota* and the order *Bacteroidales*. In ruminants, this phylum is involved in the carbohydrate and protein degradation mechanism [75,76]. The differences in the abundance of the genus *UBA1711* should result in more efficient degradation of carbohydrate, protein, and non-fiber plant monosaccharides in tyrosine-supplemented nervous and calm groups [77]. Furthermore, *RUG740* is an uncultured *Clostridiales* bacterium. *Clostridiales* are the predominant microbes known to be responsible for dysfunction of amino acid metabolism, especially affecting branched chain amino acids (BCAAs), which have been proven to affect brain function, mood, and cognition in humans [78]. Tyrosine supplementation might have reduced the abundance of *RUG740* bacteria in calm sheep since tyrosine metabolites are inversely correlated with *Clostridiales* [77]. Since this study only focused on rumen microbiota, further analysis is required for a better understanding of the effect of tyrosine on behavior and intestinal bacteria. The absorption mechanism of tyrosine in the intestine also needs to be studied.

## 5. Conclusions

This study demonstrates that dietary tyrosine supplementation reduces anxiety-like behaviors in nervous Altay sheep while enhancing rumen health through increased propionic acid production and improved antioxidant capacity. Although tyrosine had minimal effects on overall rumen diversity, it influenced specific bacterial genera (*UBA1711*, *RUG740*), suggesting temperament-dependent interactions. These findings support tyrosine’s potential as a stress-reducing feed additive in intensive farming, though further research is needed to optimize its use and long-term impacts on microbial metabolism and sheep productivity.

## Figures and Tables

**Table 1:** Composition and nutrient levels of basic diet (DM basis).

Items	Content
Silage corn %	6.0
Alfalfa %	12.0
Wheat straw %	12.0
Cotton residue %	30.0
Corn %	25.4
Soybean meal %	5.2
Bran %	8.0
NaCl %	1.0
Premix %	0.4
Total %	100
Nutrients	
Digestible energy (MJ/KG)^2^	11.2
Crude protein	15.06
Neutral detergent fiber	36.03
Acid detergent fiber	25.51

**Table 2:** The effect of tyrosine on richness and indices of biodiversity (Shannon and Simpson indices) in the microbial population in the rumen fluid of Altay sheep. CON^1^ = calm sheep with control diet, TYR^1^ = calm sheep with tyrosine supplementation, CON = nervous sheep with control diet, TYR = nervous sheep with tyrosine supplementation.

Alpha Diversity Index	Calm Type		Nervous Type		SEM	*p*-Value		
	CON^1^	TYR^1^	CON	TYR		Temperament	TYR	Temperament × TYR
Richness	2501.2	2408	2785.8	2615.6	61.458	0.046	0.265	0.740
Shannon	5.341	5.226	5.680	5.566	0.099	0.182	0.776	0.783
Simpson	0.914	0.907	0.929	0.933	0.008	0.259	0.928	0.739

**Table 3:** Effect of tyrosine on relative abundance (%) of ruminal bacteria at phylum level of Altay sheep with different temperaments. CON^1^ = calm sheep with control diet, TYR^1^ = calm sheep with tyrosine supplementation, CON = nervous sheep with control diet, TYR = nervous sheep with tyrosine supplementation. Data are expressed as mean ± SEM.

Items	Calm Type		Nervous Type		SEM	*p*-Value		
	CON^1^	TYR^1^	CON	TYR		Temperament	TYR	Temperament × TYR
Relative abundance of microorganisms at phylum level (%)								
*Bacteroidota*	43.07	48.6	40.91	39.56	2.164	0.137	0.869	0.541
*Bacillota A*	35.86	33.59	35.03	41.67	1.530	0.118	0.257	0.221
*Bacillota C*	10.08	5.45	7.28	3.94	1.400	0.387	0.149	0.903
*Bacillota*	1.78	3.44	2.63	4.26	0.475	0.902	0.512	0.330
*Planctomycetota*	1.31	1.06	3.02	2.07	0.300	0.013	0.341	0.625
*Chloroflexota*	1.86	1.7	1.71	1.79	0.207	0.990	0.990	0.757
*Pseudomonadota*	1.14	1.17	1.8	1.18	0.160	0.251	0.453	0.378
*Verrucomicrobiota*	1.22	0.77	1.36	0.89	0.145	0.523	0.213	0.830

**Table 4:** Effect of tyrosine on relative abundance (%) of ruminal bacteria at genus level of Altay sheep with different temperaments. CON^1^ = calm sheep with control diet, TYR^1^ = calm sheep with tyrosine supplementation, CON = nervous sheep with control diet, TYR = nervous sheep with tyrosine supplementation.

Items	Calm Type		Nervous Type		SEM	*p*-Value		
	CON^1^	TYR^1^	CON	TYR		Temperament	TYR	Temperament × TYR
Relative abundance of microorganisms at genus level (%)								
*Cryptobacteroides*	16.75	14.37	16.60	13.96	1.484	0.931	0.442	0.968
*Prevotella*	14.41	18.30	12.50	11.57	1.986	0.300	0.742	0.551
*Limivicinus*	6.51	8.13	7.68	7.83	0.870	0.819	0.642	0.701
*Quinella*	5.41	2.02	5.32	1.18	1.200	0.851	0.142	0.879
*UBA1711*	3.64	2.80	1.62	6.19	0.634	0.545	0.109	0.025
*RUG740*	4.41	2.75	1.86	4.22	0.468	0.546	0.700	0.035
*Saccharofermentans*	2.77	2.02	2.96	4.91	0.483	0.108	0.520	0.157
*Limimorpha*	2.37	3.84	3.33	1.90	0.621	0.712	0.987	0.279
*Sodaliphilus*	1.46	2.65	2.41	1.11	0.440	0.748	0.950	0.186
*Flexilinea*	1.84	1.69	1.69	1.82	0.206	0.979	0.993	0.759
Others	35.94	34.78	38.03	37.28	1.327	0.429	0.741	0.943

**Table 5:** Effect of tyrosine on KEGG function prediction between Altay sheep with different temperaments (KEGG pathway level 1). CON^1^ = calm sheep with control diet, TYR^1^ = calm sheep with tyrosine supplementation, CON = nervous sheep with control diet, TYR = nervous sheep with tyrosine supplementation. Data are expressed as mean ± SEM.

Items	Calm Type		Nervous Type		SEM	*p*-Value		
	CON^1^	TYR^1^	CON	TYR		Temperament	TYR	Temperament × TYR
Metabolism	133,199.2	131,954.8	126,077.1	120,634.6	2269.366	0.032	0.365	0.536
Genetic information processing	52,317.47	52,991.09	50,399.99	50,487.23	662.972	0.117	0.775	0.832
Environmental information processing	33,470.16	31,483.46	31,429.53	31,432.38	664.059	0.423	0.445	0.517
Cellular processes	24,455.08	23,012.84	23,757.06	21,446.68	605.790	0.328	0.121	0.680
Organismal systems	7601.302	7549.636	7294.472	7056.052	108.048	0.061	0.449	0.607

**Table 6:** Effect of tyrosine on KEGG function prediction between Altay sheep with different temperaments (KEGG pathway level 2). CON^1^ = calm sheep with control diet, TYR^1^ = calm sheep with tyrosine supplementation, CON = nervous sheep with control diet, TYR = nervous sheep with tyrosine supplementation. Data are expressed as mean ± SEM. *: *p* < 0.05, **: *p* < 0.01.

Pathways	Calm Type		Nervous Type		SEM	*p*-Value		
	CON^1^	TYR^1^	CON	TYR		Temperament	TYR	Temp × TYR
Global and overview maps	125,658.9	124,094.7	118,649.9	112,534.1	2132.28	0.029 *	0.336	0.556
Carbohydrate metabolism	43,722.5	44,147.8	41,715.01	40,728.57	656.26	0.044 *	0.824	0.577
Energy metabolism	20,338.4	19,943.35	19,068.3	18,515.84	344.75	0.058	0.484	0.907
Lipid metabolism	9983.62	9940.1	9531.09	9162.43	152.58	0.048 *	0.484	0.58
Amino acid metabolism	32,456.23	31,725.05	30,432.46	28,875.08	625.27	0.056	0.347	0.731
Metabolism of other amino acids	9449.35	9584.69	8920.58	8816.18	151.56	0.038 *	0.958	0.681
Glycan biosynthesis and metabolism	13,990.73	14,617.92	13,203.62	12,231.99	319.66	0.009 **	0.752	0.155
Metabolism of terpenoids and polyketides	5501.22	5344.6	5061.04	4871.98	94.32	0.014	0.312	0.923
Folding, sorting, and degradation	10,196.51	10,161.66	9669.84	9615.84	125.86	0.039	0.855	0.969
Cellular community (eukaryotes)	14.18	4.61	19.01	10.18	2.01	0.154	0.018	0.917

**Table 7:** Effect of tyrosine on KEGG function prediction between Altay sheep with different temperaments (KEGG pathway level 3). CON^1^ = calm sheep with control diet, TYR^1^ = calm sheep with tyrosine supplementation, CON = nervous sheep with control diet, TYR = nervous sheep with tyrosine supplementation.

Pathways	Calm Type		Nervous Type		SEM	*p*-Value		
	CON^1^	TYR^1^	CON	TYR		Temperament	TYR	Temp × TYR
Metabolic pathways	123,510.76	122,158.84	116,671.92	110,787.2	2075.331	0.028	0.35	0.555
Biosynthesis of secondary metabolites	55,713.56	54,795.24	52,161.55	59,868.52	1044.726	0.047	0.426	0.731
Biosynthesis of antibiotics	37,008.85	36,082.38	24,559.97	32,960.55	662.321	0.037	0.317	0.787
Carbon metabolism	16,390.99	16,117.45	15,442.19	14,895.3	243.168	0.026	0.367	0.761
Amino sugar and nucleotide sugar metabolism	10,932.43	11,056.11	10,375.66	9966.83	172.297	0.016	0.646	0.395
Two-component system	11,465.86	10,711.98	10,843.11	9881.02	228.17	0.095	0.052	0.803
Thyroid hormone synthesis	572.7	580.77	513.32	506.94	13.891	0.018	0.974	0.778
Oxidative phosphorylation	4462.99	4575.76	4160.62	3990.58	95.025	0.02	0.869	0.421
Tyrosine metabolism	685.31	632.4	681.93	639.15	19.366	0.967	0.254	0.902
Phenylalanine, tyrosine, and tryptophan biosynthesis	4425.01	3988.04	3955.29	3646.95	131.691	0.126	0.158	0.801

## Data Availability

All data are contained within this manuscript and Supplementary Materials.

## Supplementary Materials

The following supporting information can be downloaded at https://www.mdpi.com/article/10.3390/vetsci12080684/s1: Table S1: ADG and FCR in different treatment groups; Table S2: Number of crosses in arena test; Table S3: Volatile fatty acids present in rumen fluid; Table S4: Tyrosine content in the rumen fluid; Table S5: Antioxidant properties present in rumen epithelium; Table S6: Relative abundance of microbes present in rumen fluid (%); Table S7: KEGG pathway analysis

## Abbreviations

The following abbreviations are used in this manuscript:

TYR	Tyrosine
TH	Tyrosine hydroxylase
DA	Dopamine
NE	Norepinephrine
KEGG	Kyoto Encyclopedia of Genes and Genomes
SOD	Superoxide dismutase
CAT	Catalase
ROS	Reactive oxygen species
GSH	Glutathione
GSH-Px	Glutathione peroxide
MDA	Malondialdehyde
T-AOC	Total antioxidant capacity
FCR	Feed conversion ratio
ADG	Average daily gain
