# Supplementary material for: Effect of Dietary Tyrosine on Behavior and Ruminal Meta-Taxonomic Profile of Altay Sheep with Different Temperaments
Source: Vet Sci. 2025 Jul 22;12(8):684. doi: 10.3390/vetsci12080684 (PMC12389934; doi:10.3390/vetsci12080684)
Supplement: Supplementary file 1 [file vetsci-12-00684-s001.zip › Supplementary Table S1.pdf]

ADG and FCR in different treatment groups

Groups	ADG	FCR
calm	208.15	6.4
calm	210.56	5.1
calm	220.15	4.5
calm	215.56	7.1
calm	217.45	8.5
calm	212.78	6.2
calm tyrosine	245.67	5.45
calm tyrosine	250.98	4.23
calm tyrosine	255.01	6.22
calm tyrosine	260.87	7.12
calm tyrosine	265.42	8.41
calm tyrosine	253.43	6.71
nervous	242.12	6.76
nervous	240.56	7.45
nervous	235.87	8.34
nervous	245.91	5.45
nervous	141.23	4.35
nervous	244.01	6.36
nervous tyrosine	260.53	6.68
nervous tyrosine	265.75	5.87
nervous tyrosine	255.81	7.87
nervous tyrosine	268.43	4.56
nervous tyrosine	252.89	8.56
nervous tyrosine	161.78	6.68
