# Supplementary material for: Effect of Dietary Tyrosine on Behavior and Ruminal Meta-Taxonomic Profile of Altay Sheep with Different Temperaments
Source: Vet Sci. 2025 Jul 22;12(8):684. doi: 10.3390/vetsci12080684 (PMC12389934; doi:10.3390/vetsci12080684)
Supplement: Supplementary file 1 [file vetsci-12-00684-s001.zip › Supplementary Table S2.pdf]

Number of crosses in arena test

Groups	first arena test	second arena test
calm	4	6
calm	3	5
calm	2	4
calm	5	7
calm	6	8
calm	4	6
calm tyrosine	3	7
calm tyrosine	2	8
calm tyrosine	1	9
calm tyrosine	4	6
calm tyrosine	5	5
calm tyrosine	3	7
nervous	10	16
nervous	11	15
nervous	12	14
nervous	9	17
nervous	8	18
nervous	10	16
nervous tyrosine	12	8
nervous tyrosine	13	9
nervous tyrosine	14	10
nervous tyrosine	11	7
nervous tyrosine	10	6
nervous tyrosine	12	8
