# Supplementary material for: Effect of Dietary Tyrosine on Behavior and Ruminal Meta-Taxonomic Profile of Altay Sheep with Different Temperaments
Source: Vet Sci. 2025 Jul 22;12(8):684. doi: 10.3390/vetsci12080684 (PMC12389934; doi:10.3390/vetsci12080684)
Supplement: Supplementary file 1 [file vetsci-12-00684-s001.zip › Supplementary Table S3.pdf]

Volatile fatty acids present in rumen fluid

Groups	propionate	acetate	total volatile fatty acids	butyrate	A/P
calm	6.2	31.01	57.21	3.1	5.001613
calm	5.1	32.22	58.67	4.2	6.317647
calm	8.01	33.33	59.67	5.3	4.161049
calm	7.01	35.1	60.21	6.01	5.007133
calm	7.5	36.3	61.23	7.2	4.84
calm	9.02	37.21	62.11	5.05	4.125277
calm tyrosine	14.01	35.21	60.13	4.1	2.513205
calm tyrosine	13.3	36.21	61.55	5.2	2.722556
calm tyrosine	11.2	37.21	62.13	6.1	3.322321
calm tyrosine	10.1	38.01	63.11	7.2	3.763366
calm tyrosine	9.6	38.3	64.32	8.1	3.989583
calm tyrosine	8.9	38.12	64.52	6.2	4.283146
nervous	5.01	32.11	56.22	3.21	6.409182
nervous	6.21	33.13	57.11	4.1	5.334944
nervous	7.21	34.01	58.56	5.3	4.71706
nervous	8.55	35.13	59.21	6.3	4.108772
nervous	9.01	36.1	60.11	7.1	4.006
nervous	10.21	37.32	61.21	5.21	3.65524
nervous tyrosine	8.12	39.63	60.13	3.22	4.880542
nervous tyrosine	9.23	38.61	61.23	4.15	4.183099
nervous tyrosine	10.43	37.16	62.51	5.6	3.5628
nervous tyrosine	11.01	40.12	63.23	6.9	3.64396
nervous tyrosine	12.2	41.23	64.12	7.1	3.379508
nervous tyrosine	13.1	42.23	65.23	5.3	3.223664
