# Supplementary material for: Effect of Dietary Tyrosine on Behavior and Ruminal Meta-Taxonomic Profile of Altay Sheep with Different Temperaments
Source: Vet Sci. 2025 Jul 22;12(8):684. doi: 10.3390/vetsci12080684 (PMC12389934; doi:10.3390/vetsci12080684)
Supplement: Supplementary file 1 [file vetsci-12-00684-s001.zip › Supplementary Table S4.pdf]

Tyrosine content in the rumen fluid

**Groups**	**Tyrosine content**
calm	14.19367
calm	2.9341
calm	3.337777
calm	27.30325
calm	3.32033
calm	18.3785
calm tyrosine	1.453845
calm tyrosine	9.186609
calm tyrosine	4.457892
calm tyrosine	2.845426
calm tyrosine	2.879359
calm tyrosine	10.37923
nervous	14.02081
nervous	11.92015
nervous	23.20646
nervous	41.47868
nervous	7.632085
nervous	16.51842
nervous tyrosine	37.13251
nervous tyrosine	43.75908
nervous tyrosine	43.06761
nervous tyrosine	22.56541
nervous tyrosine	13.47548
nervous tyrosine	8.227036
