# Supplementary material for: Effect of Dietary Tyrosine on Behavior and Ruminal Meta-Taxonomic Profile of Altay Sheep with Different Temperaments
Source: Vet Sci. 2025 Jul 22;12(8):684. doi: 10.3390/vetsci12080684 (PMC12389934; doi:10.3390/vetsci12080684)
Supplement: Supplementary file 1 [file vetsci-12-00684-s001.zip › Supplementary Table S5.pdf]

Antioxidant properties present in rumen epithelium

Groups	CAT value	SOD value	MDA	T-AOC	GSH	GSH-Px
calm	31.11	120.98	3.78	0.88	2.6	45.2
calm	31.57	123.65	2.34	0.95	2.0	50.24
calm	33.24	125.9	1.55	0.89	2.9	49.7
calm	32.42	120.98	1.98	1.53	3.2	55.89
calm	31.98	124.32	2.45	1.76	3.5	62.56
calm	32.76	132.76	2.87	1.32	2.5	50.45
calm tyrosine	35.32	133.97	2.65	1.32	3.6	48.2
calm tyrosine	33.77	135.21	2.43	0.8	2.8	52.34
calm tyrosine	37.09	114.87	1.54	0.99	2.7	55.23
calm tyrosine	34.65	130.81	1.65	1.53	3.0	45.91
calm tyrosine	35.07	128.03	2.43	1.5	2.8	53.67
calm tyrosine	33.43	132.65	2.08	0.98	2.7	60.76
nervous	12.43	78.99	4.01	0.87	2.1	65.21
nervous	13.21	80.87	4.88	0.67	1.8	55.34
nervous	11.99	85.21	4.98	0.89	1.9	61.7
nervous	10.67	67.89	3.2	0.98	2.0	57.81
nervous	11.03	71.22	4.23	1.02	1.5	70.43
nervous	14.67	83.65	3.41	1.21	1.7	52.34
nervous tyrosine	19.89	105.1	4.03	1.32	1.9	59.41
nervous tyrosine	18.45	114.87	3.55	1.25	2.2	67.98
nervous tyrosine	16.87	113.76	2.32	1.4	2.0	63.56
nervous tyrosine	16.01	116.87	4.14	0.98	1.9	58.65
nervous tyrosine	20.03	109.7	1.34	1.88	2.5	52.98
nervous tyrosine	17.54	121.8	2.11	1.54	3.0	51.23
