# Supplementary material for: Effect of Dietary Tyrosine on Behavior and Ruminal Meta-Taxonomic Profile of Altay Sheep with Different Temperaments
Source: Vet Sci. 2025 Jul 22;12(8):684. doi: 10.3390/vetsci12080684 (PMC12389934; doi:10.3390/vetsci12080684)
Supplement: Supplementary file 1 [file vetsci-12-00684-s001.zip › Supplementary Table S6.pdf]

Relative abundance of microbes present in rumen fluid (%)

**Genus (%)**	**calm**	**calm tyrosine**	**nervous**	**nervous tyrosine**
Cryptobacteroides	16.71	34.73	16.54	12.94
Prevotella	14.45	18.37	12.53	13.62
Limivicinus	6.51	8.09	7.7	7.86
Quinella	5.44	2	5.39	1.35
UBA 1711	3.64	2.82	1.62	5.52
RUG740	4.4	2.76	1.86	4.41
Saccharofermentans	2.71	2.02	2.96	4.57
Limimorpha	2.37	3.86	3.36	1.92
Sodaliphilus	1.46	2.66	2.42	1.14
Flexilinea	1.84	1.68	1.68	1.77
Others	35.93	34.73	38	36.96
**Phylum (%)**	**Calm**	**calm tyrosine**	**nervous**	**nervous tyrosine**
Bacteroidota	43.07	48.6	40.91	39.56
Bacillota_A	35.86	33.59	35.03	41.67
Bacillota_C	10.08	5.45	7.28	3.94
Bacillota	1.78	3.44	2.63	4.26
Planctomycetota	1.31	1.06	3.02	2.07
Chloroflexota	1.86	1.7	1.71	1.79
Pseodomonadota	1.14	1.17	1.8	1.18
Verrucomicrobiota	1.22	0.77	1.36	0.89
Others	3.68	4.21	6.26	4.64
