# Supplementary material for: Effect of Dietary Tyrosine on Behavior and Ruminal Meta-Taxonomic Profile of Altay Sheep with Different Temperaments
Source: Vet Sci. 2025 Jul 22;12(8):684. doi: 10.3390/vetsci12080684 (PMC12389934; doi:10.3390/vetsci12080684)
Supplement: Supplementary file 1 [file vetsci-12-00684-s001.zip › Supplementary Table S7.pdf]

KEGG pathway analysis

KEGG level 1					
**Groups**	**Metabolism**	**genetic information processing**	**environmental information processing**	**cellular processes**	**organismal systems**
C1	150952.2	53818.97	36729.28	28890.52	8402.97
C2	137487.2	56071.54	35002.99	24863.17	7713.6
C3	130128.8	53737.71	34317.25	23741.72	7636.07
C4	119984.1	47930.06	27893	21299.44	6938.85
C5	127442.9	50029.36	33408.38	23480.54	7315.08
CT1	126605	49515.35	36359.88	27375.29	7371.82
CT2	121376.6	55523.21	32503.16	21574.86	7429.25
CT3	138053.1	53846.74	31553.25	22238.36	7620.65
CT4	129345.2	53667.03	29530.8	22598.97	7556.46
CT5	144394.4	52403.52	27470.3	21276.48	7770.07
N1	136674	54095.13	34237.84	28721.47	8225.01
N2	127686.7	49730.56	32107.87	22236.54	6875.85
N3	117060.9	46109.12	27544.21	20594.66	6813.13
N4	130985.3	52843.99	33548.5	25068.61	7620.36
N5	117979	49221.64	29709.64	22164.01	6937.72
NT1	123801.9	55739.03	33836.97	22909.19	7496.81
NT2	121684.7	50068.91	31332.97	22865.29	7078.82
NT3	115685.9	50201.29	28670.64	18965.61	6909.37
NT4	109524.7	47577.36	28284.96	19491.95	6445.71
NT5	127243.9	48906.77	34195.94	22364.15	7202.08

KEGG Level 2										
Groups	1.0 Global and overview maps	1.1 Carbohydrate metabolism	1.2 Energy metabolism	1.3 Lipid metabolism	1.5 Amino acid metabolism	1.6 Metabolism of other amino acids	1.7 Glycan biosynthesis and metabolism	1.9 Metabolism of terpenoids and polyketides	2.3 Folding, sorting and degradation	4.3 Cellular community - eukaryotes
C1	141816.7	46279.19	22463.04	10281.71	38026.99	9489.12	15599.68	5948.85	10746.38	14512.36
C2	129888.2	45457.84	21360.92	10931.67	33459.84	9938.22	14016.02	5827.96	10770.22	14661.18
C3	123174.7	43987.2	19428.56	10049.7	30553.54	9935.47	14466.05	5689.13	10382.53	13520.82
C4	113165	39610.37	18712.06	9033.48	29371.51	8652.48	12652.5	4838.99	9205.54	11823.04
C5	120250.1	43277.7	19726.65	9621.54	30869.28	9231.51	13219.44	5201.2	9877.9	13074.05
CT1	119180.7	40961.75	18646.84	8893.04	31109.16	8630.35	12837.89	5211.98	9577.45	14945.7
CT2	114199.4	41389.34	19054.44	9290.54	28347.08	8846.42	13044.48	5093.85	10391.37	12919.34
CT3	129594.9	45936.86	21063.94	10623.59	33100.04	10352.94	15842.74	5589.25	10366.92	12748.58
CT4	121881.5	42923.29	19937.89	9987.96	31872.06	9586.1	14375.92	5266.95	10168.72	12365.51
CT5	135617.1	49527.77	21013.69	10905.39	34196.96	10507.66	16988.58	5561.01	10303.85	11272.24
N1	128567	43442.78	21379.64	9501.04	34143.68	9043.51	14222.18	5183.73	10348.31	14430.99
N2	120143	44292.41	18888.42	9605.99	30001.11	8836.01	13820.61	5281.03	9984.76	12701.91
N3	110011.2	38985.04	17035.91	9321.99	27991.34	8576.66	12710.98	4579.86	8742	11319.3
N4	123443.3	42940.96	20596.01	9894.28	31700.93	9258.47	13066.49	5499.52	9988.2	13888.39
N5	111085	38913.92	17441.56	9332.17	28325.28	8888.26	12197.88	4761.06	9285.95	12751.9
NT1	116754.6	41922.76	19638.83	9964.69	29901.06	9621.37	11676.08	5443.76	10114.62	14106.3
NT2	114233.7	41420.01	18183.33	9444.92	28081.28	8629.2	13094.68	4902.96	9740.16	12582.73
NT3	108765.7	39840.82	17845.51	8625.94	28734.49	8575.23	12490.47	4476.23	9742.27	11358.56
NT4	102973.9	37471.02	16985.57	8512.38	25974.79	7782.94	11275.98	4625.14	8980.75	11425.79
NT5	119942.6	42988.24	19925.97	9264.22	31683.81	9472.17	12622.74	4911.83	9501.42	13663.7

KEGG level 3																
**Groups**	**Metabolic pathways**	**Biosynthesis of secondary metabolites**	**Biosynthesis of antibiotics**	**Microbial metabolism in diverse environments**	**Carbon metabolism**	**Amino sugar and nucleotide sugar metabolism**	**Two-component system**	**Tyrosine metabolism**	**Thyroid hormone synthesis**	**Starch and sucrose metabolism**	**Oxidative phosphorylation**	**Oxytocin signaling pathway**	**Dopaminergic synapse**	**Degradation of aromatic compounds**	**Phenylalanine, tyrosine and tryptophan biosynthesis**	**Phenylalanine metabolism**
C1	139024.1	65011.67	42513.48	30507.35	17382.32	10856.96	12925.37	865.2492	535.1727	7403.346	4967.148	12.44603	3.837429	823.5939	5922.277	1434.926
C2	127714.6	57515.4	38527.5	29260.46	17400.33	11418.18	11129.99	731.1133	598.1794	7183.107	4638.245	1.270004	2.417409	807.2898	4432.728	1396.423
C3	121109	53567.83	35705.47	26783.49	16167.48	11397.79	11473.66	593.7733	539.8519	7022.936	4201.279	1.187536	3.79515	668.39	3995.539	1141.579
C4	111354.1	49031.31	32959.09	24795.27	15167.93	10249.19	9984.173	568.8098	640.1607	5846.709	4195.91	2.348717	4.039257	680.0434	3634.33	1108.199
C5	118352	53441.63	35338.74	26559.68	15836.92	10740.05	11816.13	667.6088	550.1578	7134.843	4312.414	9.309192	4.93815	700.4744	4140.221	1220.021
CT1	117157.4	52807.47	34657.96	26745.75	15202.46	10263.97	11817.49	730.6946	489.5242	6642.528	4017.807	0.999589	7.878915	873.9666	3903.792	1279.327
CT2	112620.2	49979.47	33162.01	25633.3	15357.54	10150.4	9817.777	655.2373	542.2074	7386.052	4126.11	2.466233	1.160309	658.4202	3361.622	1093.748
CT3	127570.6	56858.08	37564.24	27513.6	16838.92	11527.87	10911.01	565.2613	620.5773	7424.121	5109.821	0.413648	2.077504	653.8687	4143.383	1201.913
CT4	119887	53640.4	36146.12	26650.17	16416.4	10803.47	10478.51	607.3561	611.4809	6608.973	4423.466	1.791112	1.828589	667.1495	3991.107	1166.508
CT5	133559	60690.81	38881.6	27636.55	16771.96	12534.85	10535.15	603.4528	640.07	8094.545	5201.636	0.66655	0.918043	701.8091	4540.301	1289.117

									7			2				
N1	126216.2	56770.03	37821.13	29241.64	16795.84	10833.15	12335	803.3435	543.804	6762.885	4479.719	5.920081	1.983766	770.9114	4545.592	1375.511
N2	118284.1	52719.81	34804.81	26243.93	15460.74	10930.23	10928.07	670.7967	471.2868	7242.796	4078.464	15.21561	6.61893	623.2449	3966.406	1184.185
N3	108275.2	47804.18	31409.87	23678.31	13962.47	9785.119	9936.166	546.568	508.3434	6051.22	3814.078	15.33537	4.757626	638.5747	3601.513	1105.142
N4	121236.6	54826.17	36467.79	27952.03	16439.92	10497.18	11225.7	770.3548	513.6823	6838.234	4531.242	5.701784	4.386871	779.9874	4267.239	1340.323
N5	109347.4	48687.59	32296.24	24532.35	14552.01	9832.641	9790.654	618.6002	529.5031	6142.398	3899.631	1.697031	5.992858	706.8014	3395.742	1125.919
NT1	114983.6	51817.13	34469.87	26773.87	15930.36	10526.53	9859.91	674.8609	635.3909	7089.891	4028.657	15.31682	8.122245	680.4888	3575.271	1210.856
NT2	112310.9	50111.74	33064.71	25499.34	14711.49	10519.36	10483.47	555.0095	446.9287	6712.678	3971.29	1.720467	3.882995	643.2817	3698.582	1027.036
NT3	107152.2	48860.8	32180.81	23408.97	14008.94	9605.772	9183.695	611.0679	464.3841	7376.5	3980.905	10.32168	0.466811	581.3467	3648.753	1109.493
NT4	101435.8	45229.82	30029.08	22819.62	14008.76	9173.641	9063.554	655.7121	465.6961	6367.962	3635.714	0.520191	1.993365	578.8746	3268.729	1042.014
NT5	118053.5	53323.12	35060.29	26823.07	15817	10008.88	10814.51	699.1117	522.3147	7385.679	4336.365	4.875465	8.782968	689.0397	4043.453	1303.645

C represents calm group, CT represent calm sheep with tyrosine treatment, N represent nervous group and NT represent nervous sheep with tyrosine treatment.
